# Hamming Distance Optimized Underwater Acoustic OTFS-IM Systems

**DOI:** 10.3390/e25070972

**Published:** 2023-06-24

**Authors:** Xiaopeng Guo, Biao Wang, Yunan Zhu, Zide Fang, Zhaoyue Han

**Affiliations:** Ocean College, Jiangsu University of Science and Technology, Zhenjiang 212100, China

**Keywords:** underwater acoustic communication, OTFS, index modulation, hamming distance

## Abstract

The orthogonal time frequency space (OTFS) modulation technique can provide reliable communication in time-varying channels. Due to the dispersive characteristics of underwater acoustic channels, this paper proposes an OTFS-IM underwater acoustic communication system based on Hamming distance optimization to reduce the impact of dispersion in underwater acoustic communication. Firstly, the OTFS-IM underwater acoustic communication system is introduced, which introduces index modulation into the Delay–Doppler (DD) domain to make the OTFS system have stronger anti-Delay–Doppler capability. In contrast, since there is index sequence redundancy in a specific index combination, a Hamming distance optimization model is used to eliminate the redundant combination in the specific index combination sequence and further improve the bit error rate performance of the system. In addition, the Hamming distance optimized OTFS-IM underwater acoustic communication system is verified by simulation analysis. The results show that the proposed Hamming distance optimized OTFS-IM can achieve more reliable bit error rate performance.

## 1. Introduction

Underwater acoustic channels pose inevitable challenges to the stability, reliability, and transmission rate of underwater acoustic communication systems due to their complex multipath effects, time-varying effects, and limited bandwidth [1,2], Multi-carrier modulation technology is widely used in the field of underwater acoustic communication by virtue of its strong anti-interference ability and high spectral efficiency [3], of which orthogonal frequency division multiplexing (OFDM) [4] is a typical representative of multi-carrier modulation technology that is the focus of research in underwater acoustic communication, and with the continuous updating of multi-carrier modulation technology, filter bank multi-carrier (FBMC) [5,6] modulation, orthogonal time frequency spatial (OTFS) [7,8,9,10,11] modulation, and other multi-carrier modulation technology have emerged and become the research focuses of communication technology.

Under the condition of fast movement, the channel presents fast time-varying fading characteristics, at which time the orthogonality between OFDM subcarriers is destroyed. In this case, Hadani R [7] first proposed OTFS modulation technology, which provides strong Delay and Doppler elasticity by modulating information in the DD domain. A series of extensions of OTFS technology have also been proposed. In [9], the author analyzes the performance of coded OTFS on high mobility channels and proves the basic trade-off between the coding gain and diversity gain of the system. Capable of providing stable communication in time-varying environments, the time-varying and strong Doppler effects of the underwater acoustic channel fit well with the characteristics of OTFS, making OTFS modulation techniques a hot research topic in underwater acoustic communications [12,13,14,15,16]. In [12], OTFS modulation technology was first applied to the field of underwater acoustic communication, and the performance of OTFS in underwater acoustic communication was verified by bit error rate (BER), spectrum effectiveness (SE), and peak to average power rate (PAPR). Subsequently, in the context of multi-user underwater acoustic communication, BOCUS M J [13] combined OTFS modulation techniques with MIMO to investigate a large-scale MIMO–OFDM-based OTFS multi-user underwater acoustic communication scheme. At the receiving end of the system, Ref. [14] proposed an optimal coordinate descent infinite parametric constrained equilibrium algorithm for the problem of high complexity of the linear minimum mean square error (LMMSE) algorithm, which reduces the complexity of the system while ensuring the BER performance of the system. In contrast, Ref. [15] also proposed a two-dimensional passive time-reversal receiver to reduce the complexity of the algorithm at the receiver side of the system and verified the proposed algorithm in simulated and measured channels to obtain a better system BER performance with lower complexity. Ref. [16] proposed a method of Doppler compensation for OTFS systems that mitigates the severe Doppler effect in underwater acoustic communication systems and avoids problems caused by fractional Doppler shifts through the design of channel estimation. With continuous research on multi-carrier modulation techniques, inspired by the orthogonal frequency division multiplexing with index modulation (OFDM-IM) [17] technique, the [18] combined index modulation techniques with OTFS to propose orthogonal time frequency space with index modulation (OTFS-IM), which transmits information through constellation symbols and index bits in the DD domain, achieves better BER performance at high spectral efficiency compared to the OFDM-IM system. At the same time, the addition of index bits improves the spectral efficiency and system performance of the OTFS system.

A series of indexed modulation techniques such as OFDM-IM are widely used in underwater acoustic communications [19,20,21]. However, it is difficult to realize robust work in the face of a strong Doppler environment in the time-varying underwater acoustic channel, and it is limited in a series of dynamic underwater acoustic communication scenarios. The OTFS-IM modulation technique is based on the OTFS modulation technique and combined with index modulation, which inherits the characteristic that OTFS has of a strong anti-Doppler effect in DD domain. Additionally, because the addition of index modulation enables the OTFS-IM system to have more flexible and controllable SE and BER than the OTFS system, it can achieve a better compromise between transmission reliability and SE in time-varying channels, but OTFS-IM underwater acoustic communication systems are just starting.

This paper constructs the OTFS-IM underwater acoustic communication system and proposes a Hamming distance optimization model for the problem of redundancy in activator grid combinations generated by the specific index combination method at the transmitter side of the system. Encoding the activation states of sub-grid groups, using the Hamming distance between the encoded and activated sub-grid combinations, and selecting the sequence of index combinations with a maximum Hamming distance for modulation avoids redundant combinations in a particular index combination. In contrast, the maximum likelihood (ML) detection algorithm was used on the receiver side to verify against the greedy detection (GD) detection algorithm.

## 2. OTFS-IM for Underwater Acoustic Communication System

### 2.1. System Model

**Figure 1:**
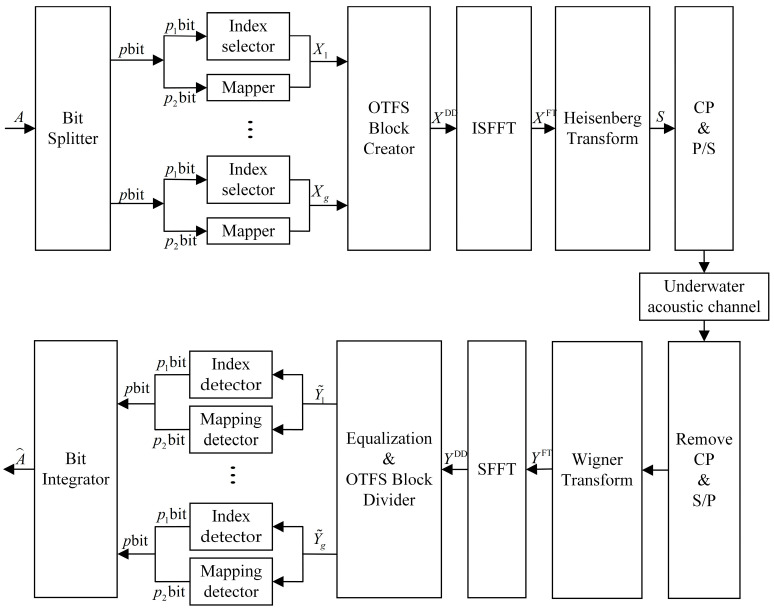
Block diagram of the underwater acoustic OTFS-IM system.

The OTFS-IM system improves the spectral efficiency of the system by making the signal transmitted in the DD domain carry a portion of the index bits through index modulation. When defining an OTFS data frame size as M×N, a data frame occupies a duration of NT and a bandwidth of B=MΔf while Δf denotes the subcarrier interval and T=1/Δf denotes the duration of each group of symbols. As shown in Figure 1, the A bit information is divided into g groups with p=A/g bit information in each group by a bit splitter at the system transmitter, and the OTFS data frames in the corresponding DD domain are also divided into g grid groups. The size of each DD domain grid group is n=MN/g. The p bit information of each data group is divided into two parts: $p_{1}$ bit and $p_{2}$ bit. $p_{1}$ bit is the index bit used to index the position of the corresponding active sub-grid in the DD domain sub-grid group. When using k to denote the number of grids in a sub-grid group that are activated by a sub-grid, a total of $C_{n}^{k}$ (C is a binomial coefficient) seed grid activation combinations are available, and the number of bits transmitted by the index selector can be expressed as:

(1) $$p_{1}=\left\lfloor \log_{2}C_{n}^{k}\right\rfloor$$

$\left\lfloor \cdot \right\rfloor$ is rounded down. Taking the βth group of the sub-grids as an example, and selecting k sub-grids from the n sub-grids to activate the bearer information, the index selector output can be expressed as:

(2) $$I_{\beta }=\left\{i_{\beta ,1},i_{\beta ,2},\cdots ,i_{\beta ,k}\right\}$$

where $i_{\beta ,l}\in \left[1,2,\cdots ,n\right]$, β=1,2,⋯,g, l=1,2,⋯,k. The other part of the $p_{2}$ bit is used for the traditional constellation symbol mapping and can be represented as:

(3) $$p_{2}=k\log_{2}W$$

W represents the dimension of constellation mapping, and the output of constellation mapper in group β can be expressed as:

(4) $$S_{\beta }=\left\{s_{\beta ,1},s_{\beta ,2},\cdots ,s_{\beta ,k}\right\}$$

where $s_{\beta ,\alpha }\in W$,β=1,2,⋯g,α=1,2,⋯,k.The constellation symbols of $S_{\beta }$ are mapped correspondingly according to the position of the activated sub-grid selected by $I_{\beta }$. The data output from the βth sub-grid groups after the index mapping can be expressed as:

(5) $$X_{\beta }={\left[x_{\beta }\left(1\right),x_{\beta }\left(2\right),\cdots ,x_{\beta }\left(n\right)\right]}^{T}$$

(6) $$x_{\beta }\left(\gamma \right)=\left\{\begin{array}{c} \begin{array}{cc} s_{\beta ,\gamma }, & \gamma \in I_{\beta } \end{array}\\ \begin{array}{cc} 0, & \gamma \notin I_{\beta } \end{array} \end{array}\right.$$

After completing index modulation for each sub-grid group in the DD domain, $X_{\beta }$,β=1,2,⋯,g is obtained. After the OTFS block generator, the DD-domain signal is the output, and after the inverse symplectic Fourier transform (ISFFT), the time frequency domain signal is obtained:

(7) $$X^{FT}=F_{M}X^{DD}F_{N}^{H}$$

$F_{M}$ and $F_{N}$ are the Fourier transform matrices, and the superscript H is the conjugate matrix. The time frequency domain signal is obtained in the time domain by the Heisenberg transform:

(8) $$S=F_{M}^{H}X^{FT}=X^{DD}F_{N}^{H}$$

Finally, to avoid inter-symbol interference (ISI) between the OTFS blocks, a cyclic prefix (CP) is added to each data frame to obtain the complete transmit signal.

### 2.2. Underwater Acoustic Channel

The fading time-varying underwater acoustic channel model can be approximated by a time-delay Doppler double extension function. In the time-delay Doppler domain, the double-extended channel function has the characteristics of two-dimensional sparsity. In this paper, we adopt the channel based on the statistical model proposed in [22], and the transfer function of the time-varying multipath underwater acoustic channel can be expressed as:

(9) $$H\left(f,t\right)={\bar{H}}_{0}\sum_{p}h_{p}{\tilde{\gamma }}_{p}\left(f,t\right)e^{-j2\pi f\tau_{p}}$$

where ${\tilde{\gamma }}_{p}\left(f,t\right)=\gamma_{p}\left(f,t\right)e^{j2\pi a_{p}ft}$ represents the overall small-scale path coefficient and p, $\tau_{p}$, $h_{p}$, and $a_{p}$ represent the number of paths, delay of each path, path gain, and Doppler factor, respectively.

### 2.3. Detection Algorithm

As shown in Figure 1, the received signal is equalized, and CP is removed and serially transformed at the receiver end of the system and then converted to a time frequency signal by means of the Wigner transform:

(10) $$Y^{FT}=F^{M}Y^{T}$$

The DD domain received signal is then obtained by the SFFT:

(11) $$Y^{DD}=F_{M}^{H}Y^{FT}F_{N}=Y^{T}F_{N}$$

After the DD domain signal passes through channel equalization and the OTFS block divider, $Y^{DD}$ is divided into g groups for the index detector and mapping detector. The grouped signal can be expressed as:

(12) $${\tilde{Y}}^{DD}=\left[{\tilde{Y}}_{1}^{DD},\cdots ,{\tilde{Y}}_{\beta }^{DD},\cdots ,{\tilde{Y}}_{g}^{DD}\right]$$

where group β is:

(13) $${\tilde{Y}}_{\beta }^{DD}={\left[{\tilde{y}}_{\beta }^{DD}\left(1\right),{\tilde{y}}_{\beta }^{DD}\left(2\right),\cdots ,{\tilde{y}}_{\beta }^{DD}\left(n\right)\right]}^{T}$$

In signal detection, OTFS-IM differs from conventional OTFS in that OTFS-IM needs to detect the index bit information carried by the active sub-grids in the DD domain with the symbol bits. In this paper, two different detection algorithms are used.

#### 2.3.1. ML Detection Algorithms

The ML detection algorithm achieves joint detection of index positions and constellation symbols for each sub-grid group by searching for all possible combinations of indexes and constellation symbols by exhaustive enumeration. Using group β as an example:

(14) $$\left({\hat{I}}_{\beta },{\hat{S}}_{\beta }\right)=\arg\underset{I_{\beta },S_{\beta }}{\min}{\sum_{\alpha =1}^{k}\left|{\tilde{y}}_{\beta }^{DD}\left(i_{\beta ,\alpha }\right)-s_{\beta }\left(\alpha \right)\right|}^{2}$$

where $s_{\beta }\left(\alpha \right)$ denotes the α symbol in the set of legal constellation symbols. After detection, the signal ${\hat{I}}_{\beta }$ and ${\hat{S}}_{\beta }$ return the $p_{1}$ bit index information and $p_{2}$ bit symbol information through index and symbol decoding.

#### 2.3.2. GD Detection Algorithm

The GD detection algorithm is divided into two steps. Again, using group β as an example: the k active sub-grid positions in the sub-grid group are first detected, and the k most energetic sub-grids corresponding to index ${\hat{I}}_{\beta }=\left\{{\hat{i}}_{\beta ,1},{\hat{i}}_{\beta ,2},\cdots ,{\hat{i}}_{\beta ,k}\right\}$ are detected by solving for the energy of each sub-grid.

Next, ML detection is performed on the symbol information at each of the k active sub-grid locations detected in the sub-grid group.

(15) $${\hat{S}}_{\beta }=\arg\underset{S_{\beta }}{\min}\sum_{\alpha =1}^{k}{\left|{\tilde{y}}_{\beta }^{DD}\left({\hat{i}}_{\beta ,\alpha }\right)-s_{\beta }\left(\alpha \right)\right|}^{2}$$

The GD detection algorithm changes the detection method of active sub-grid index positions compared to ML detection and uses the ML detection algorithm only for the detection of constellation symbols. The GD detection algorithm is therefore a major improvement over ML detection in terms of complexity, but at the cost of a certain loss of reliability of the detection algorithm.

## 3. Hamming Distance Optimized Index Mapping Model

### 3.1. Index Mapping Method

There are two methods of mapping index bits to active sub-grid positions, including the lookup table method and the combined model method. The lookup table method involves creating an indexed mapping table of size $2^{p_{1}}$ at the sender and receiver sides and mapping the $p_{1}$ bits at the sender side to the locations of active sub-grids by looking up the table. Additionally, at the receiver side, the $p_{1}$ bits of the corresponding input are obtained by querying the location of the detected active sub-grid. Take $\left(n,k\right)=\left(4,2\right)$ as an example where $p_{1}=\left\lfloor \log_{2}C_{n}^{k}\right\rfloor =2$ and the table size is $2^{p_{1}}=4$. As shown in Table 1, when the index bit is $\left[\begin{array}{cc} 0 & 0 \end{array}\right]$, the corresponding active sub-grid position is $\left[\begin{array}{cc} 1 & 2 \end{array}\right]$, and the sub-grid group ${\left[\begin{array}{cccc} s_{\beta ,1} & s_{\beta ,2} & 0 & 0 \end{array}\right]}^{T}$ is obtained by mapping the constellation symbol to the active sub-grid. The table lookup method is simple and easy to implement when $C_{n}^{k}$ is small.

However, when $C_{n}^{k}$ is large, the size of the table also increases, making the table query more complex. The combinatorial model method solves the problem of an excessive complexity of the table lookup method when $C_{n}^{k}$ is large by the method of combinatorial numbers in mathematical calculations by converting $p_{1}$ bits into decimal numbers R where there is a one-to-one mapping relationship with k combinatorial values in the combinatorial numbers. For any R, there exists a unique decreasing sequence $K=\left\{c_{k},\cdots ,c_{1}\right\}$, $c_{k}>\cdots >c_{1}\geq 0$ of length k, such that (16) holds.

(16) $$R=C_{c_{k}}^{k}+C_{c_{k-1}}^{k-1}+\cdots +C_{c_{2}}^{2}+C_{c_{1}}^{1}$$

In the combined model approach, the maximum value of $c_{k}$ that satisfies $C_{c_{k}}^{k}\leq R$ is first chosen, followed by the maximum value of $c_{k-1}$ that satisfies $C_{c_{k-1}}^{k-1}\leq R-C_{c_{k}}^{k}$, and so on until the $c_{1}$ is finally selected and the index sequence I=K+1 corresponding to the position of the activated sub-grid is obtained from sequence K. Taking $\left(n,k\right)=\left(8,3\right)$ as an example, each sub-grid group size is set to 8, where the number of active sub-grids is 3. There are $C_{n}^{k}=56$ different ways of combining the sub-grids:

(17) $$\begin{array}{cccc} 55=C_{7}^{3}+C_{6}^{2}+C_{5}^{1} & \to & K=\left\{7,6,5\right\} & I_{\beta }=\left\{8,7,6\right\}\\ 54=C_{7}^{3}+C_{6}^{2}+C_{4}^{1} & \to & K=\left\{7,6,4\right\} & I_{\beta }=\left\{8,7,5\right\}\\ \vdots & & \vdots & \vdots \\ 1=C_{3}^{3}+C_{1}^{2}+C_{0}^{1} & \to & K=\left\{3,1,0\right\} & I_{\beta }=\left\{4,2,1\right\}\\ 0=C_{2}^{3}+C_{1}^{2}+C_{0}^{1} & \to & K=\left\{2,1,0\right\} & I_{\beta }=\left\{3,2,1\right\} \end{array}$$

### 3.2. Hamming Distance Optimization Model

In the use of combined model method and table lookup table method, when $C_{n}^{k}>2^{p_{1}}$, no matter which mapping method is used, there will be a problem as the number of generated index combination sequences is greater than the number of required index combination sequences. We call the extra index combination sequence a redundant index combination sequence. By encoding the index combination sequence according to the state of its corresponding position sub-grid, we can obtain the hamming distance between each index combination sequence and the average Hamming distance of the index combination sequence set because the larger the hamming distance is, the lower the probability of errors caused by the channel and noise in the detection of the receiver is. The existence of redundant sequences makes the average hamming distance of the index combination sequence set smaller which interferes with the BER performance of the system. We design a Hamming distance optimization model to solve the redundancy problem in index combination sequences. This model maximizes the average Hamming distance of the set by identifying and deleting redundant index combination sequences in the set. In other words, the redundant index combination sequences are deleted from the directly generated set containing $C_{n}^{k}$ index combination sequences, and an index combination sequence set consisting of $2^{p_{1}}$ required index combination sequences is obtained, and the index combination sequence set has the maximum average hamming distance.

**Figure 2:**
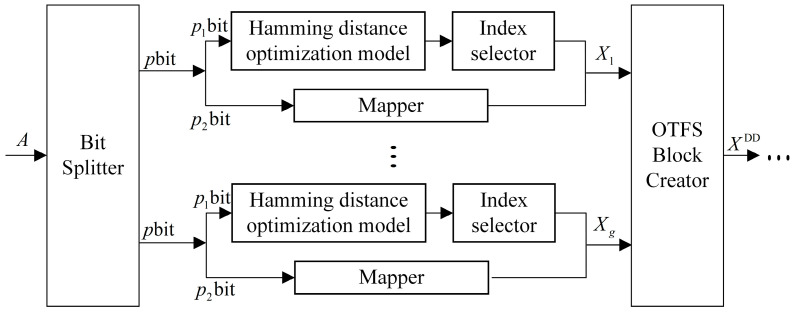
Transmitter of the Hamming distance optimization modeling system.

At $\left(n,k\right)=\left(8,3\right)$,$p_{1}=\left\lfloor \log_{2}C_{n}^{k}\right\rfloor =\left\lfloor \log_{2}C_{8}^{3}\right\rfloor =5$,$2^{p_{1}}=32$; therefore, only 32 different combinations of index sequences are needed, whereas there are $C_{n}^{k}=56$ in the index combination method, and there is considerable redundancy in the index combination sequences. This redundancy is always present when $C_{n}^{k}>2^{p_{1}}$. To further improve the performance of the system BER, a maximum Hamming distance optimization index combination model is used to eliminate redundant combinations in the index combination sequence before index modulation is performed, as shown in the Figure 2.

**Figure 3:**
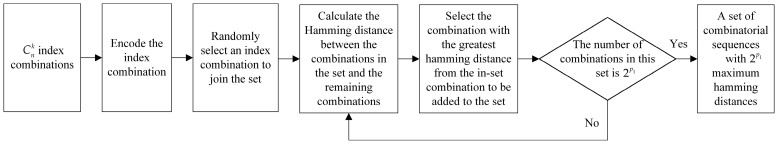
Maximum Hamming distance indexed combined sequence selection flowchart.

The selection of the maximum Hamming distance index combination sequence set is shown in Figure 3. First, the index combinations of the sub-grid groups are coded, with active sub-grids coded as 1 and inactive sub-grids coded as 0. Second, an index sequence is randomly selected from all the index combination sequences. The selected sequence is denoted as $C_{1}$. Then, the encoded hamming distance between the encoded $C_{1}$ and all the remaining index combination sequences is calculated, and the index combination sequence $C_{2}$ with the largest hamming distance from $C_{1}$ is selected. When calculating the maximum hamming distance, there may be several groups of index combination sequences with the same maximum Hamming distance as $C_{1}$. In this case, one group of index combination sequences with the maximum Hamming distance is randomly selected as $C_{2}$. Since the purpose of the Hamming distance optimization model is to remove the performance interference caused by the redundant combinations in the index combination sequence, the purpose of deleting the redundant combinations and selecting the maximum Hamming distance index combination can be achieved by this method. After determining $C_{2}$, the index combination sequence set is updated to $\left\{C_{1},C_{2}\right\}$. Then, the average hamming distance between the remaining unselected index combination sequence and the selected index combination sequence set is calculated, and the index combination sequence with the largest hamming distance from the selected index combination sequence set is selected to be added to the set. The above steps are repeated until the number of index combination sequences in the selected set is $2^{p_{1}}$; then, the set of index combination sequences with the largest Hamming distance can be obtained.

When the hamming distance between different index combinations is obtained by calculating the hamming distance between codes, the corresponding maximum Hamming distance is calculated as:

(18) $$d_{\max}\left(H\right)=\underset{2^{p_{1}}}{\max}\sum_{i=1}^{C_{n}^{k}}\sum_{j=1,j\neq i}^{C_{n}^{k}}HD\left(X_{i}^{\prime },X_{j}^{\prime }\right)$$

where $HD\left(\cdot \right)$ denotes the Hamming distance between combinations and $X_{i}^{\prime }$, $X_{j}^{\prime }$ is the encoded sub-grid group. Equation (18) allows for the selection of the $2^{p_{1}}$ combinations with the greatest Hamming distance from the $C_{n}^{k}$ index combinations to form the final sequence of index combinations at $C_{n}^{k}>2^{p_{1}}$. Taking $\left(n,k\right)=\left(4,2\right)$ as an example, all combinations of $C_{n}^{k}=6$ belong to $\left[0,C_{n}^{k}-1\right]$ and satisfy $C_{n}^{k}>2^{p_{1}}$. We need to select $2^{p_{1}}=4$ indexed combinations from 6 combinations to form the final indexed combination sequence. The four combinations with the maximum Hamming distance index were obtained by the calculation of equation (18). As shown in Table 2, the Hamming distance optimization model removes two redundant combinations from the original six index combination sequences, and the average Hamming distance of the selected combinations is 2.67, which has the maximum average Hamming distance. The greater Hamming distance between different combinations, the smaller the probability of errors during demodulation, thus achieving the purpose of improving the BER performance of the system.

## 4. Simulation Analysis

**Figure 4:**
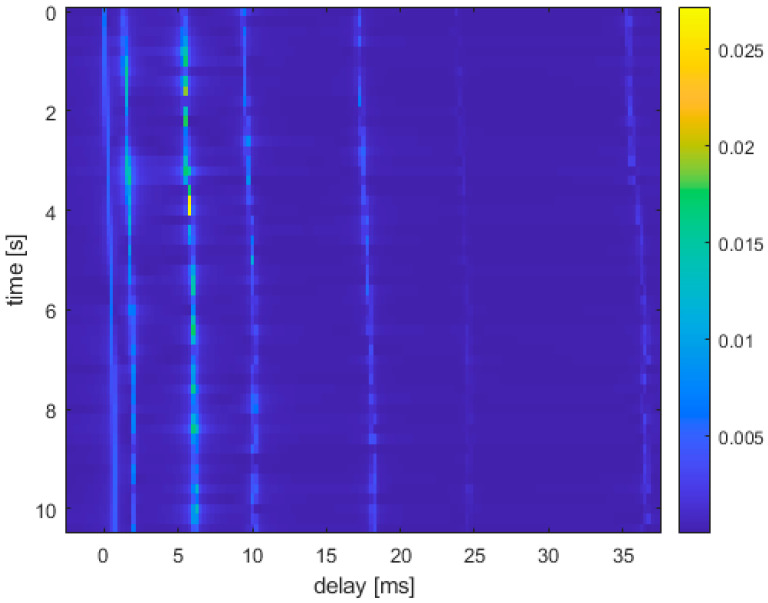
Time-varying underwater acoustic channel.

In this section, we first verify the trade-off between BER performance benefits and the spectral efficiency of OTFS-IM modulation techniques in underwater acoustic communication systems by simulations. Then, the performance gain of the Hamming distance optimization model in the OTFS-IM underwater acoustic communication system under two demodulation algorithms is analyzed, and the performance gain of the Hamming distance optimization model is verified. The channel parameters used in the simulations are shown in Table 3, and the time-varying underwater acoustic channel impulse response is shown in Figure 4.

Based on the characteristics of the time-varying underwater acoustic channel, we set the parameters of the OTFS-IM system as M=256 and N=128; the signal bandwidth is set to 4kHz; the subcarrier spacing is Δf=15.63 Hz; the symbol spacing is set to $T_{s}=0.25\;\mathrm{ms}$; and the CP prefix length is set to $T_{cp}=16\;\mathrm{ms}$. The duration of an OTFS-IM frame can therefore be expressed as $T_{OTFS-IM}=10.24\;s$. The QPSK modulation mode is used in the simulation, while the channel is assumed to be completely known at the receiver end.

The data transmitted by the OTFS-IM system consists of index bits and constellation modulation bits; therefore, the spectrum efficiency of OTFS-IM can be expressed as:

(19) $$\eta =\frac{\left\lfloor \log_{2}C_{n}^{k}\right\rfloor +k\log_{2}W}{n}$$

It can be seen that the spectral efficiency of the OTFS-IM system is related to the packet size n, the number of active sub-grids k, the constellation mapping dimension W, and other parameters.

We first simulated the BER performance of the proposed OTFS-IM underwater acoustic communication system under different spectrum efficiencies. Dual validation of the relationship between BER performance and spectrum efficiency uses two different receiver side detection algorithms—the ML detection algorithm and the GD detection algorithm—and the receiver side uses MMSE equalization to counteract the effects of the channel on the transmitted signal.

**Figure 5:**
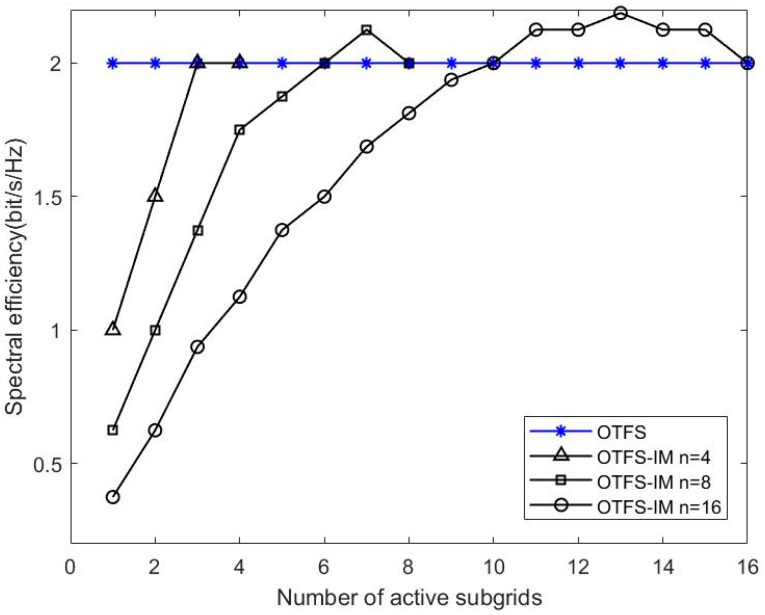
Spectral efficiency diagram for different values of $\left(n,k\right)$.

**Figure 6:**
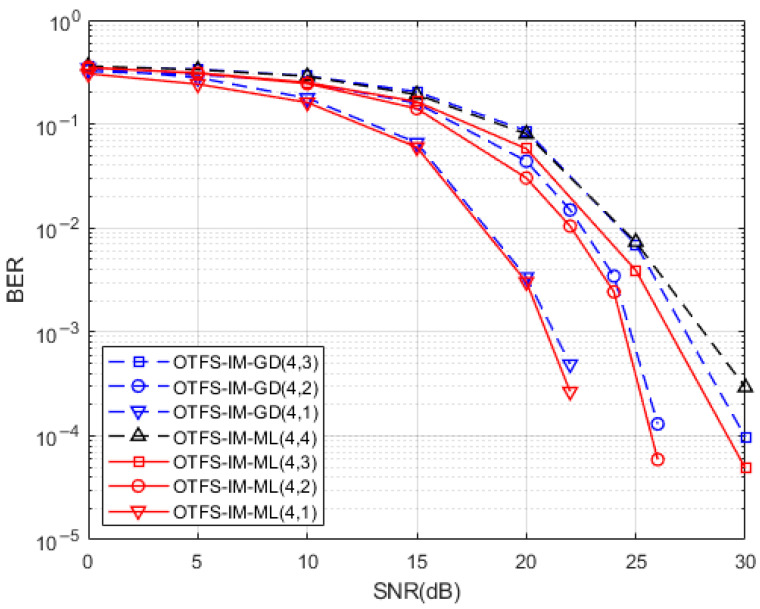
Simulation comparison of the number of different active sub-grids.

Figure 5 shows the spectral efficiency plots for different values of $\left(n,k\right)$. Figure 6 shows a comparison of the simulations of the two detection methods with different numbers of active sub-grids. The sub-grid group size is set to n=4. Three conclusions can be drawn from the combination of Figure 4 and Figure 5. Firstly, the spectrum efficiency of conventional OTFS systems can be achieved or even exceeded by means of index modulation for certain combinations of activation sub-grid numbers. At the same time, its system BER performance is better than that of conventional OTFS systems. The number of active sub-grids in the index modulated OTFS block is less than in a conventional OTFS system; therefore, there is an improvement in energy efficiency. Secondly, when comparing the BER performance of two different receiver detection algorithms with the same sub-grid group size, we found that the smaller the number of active sub-grids, the better the BER performance of the system. When the number of active sub-grids in the system sub-grid group is 2, the system BER reaches 0 after the SNR is 26dB, and when the number of active sub-grids is 1, the system BER reaches 0 after 22dB. This performance gain is obtained through a reduction in spectral efficiency so OTFS-IM systems can obtain a trade-off between system BER performance and spectrum efficiency. Finally, the BER performance at the receiver end of the system is significantly better than the GD algorithm when the ML algorithm is used for detection, but the GD detection algorithm outperforms the ML detection algorithm in terms of algorithm complexity.

**Figure 7:**
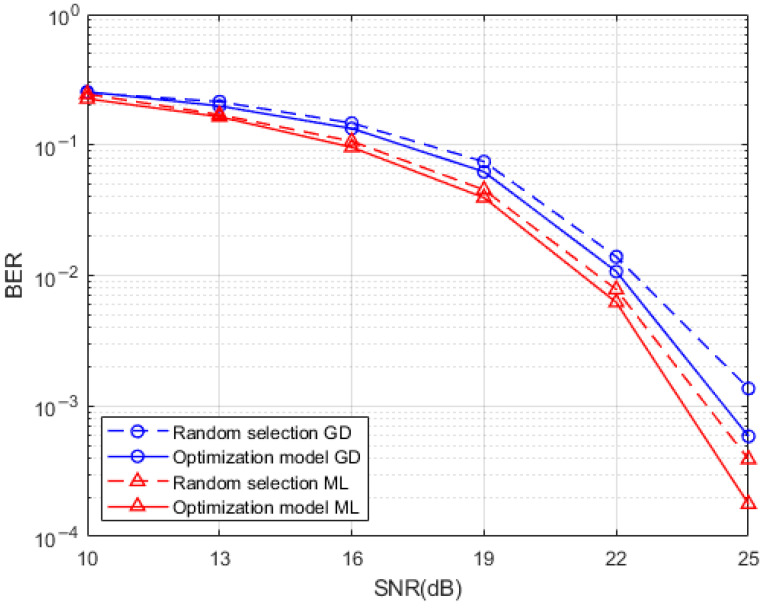
Hamming distance optimized index mapping and random selection mapping performance comparison.

**Figure 8:**
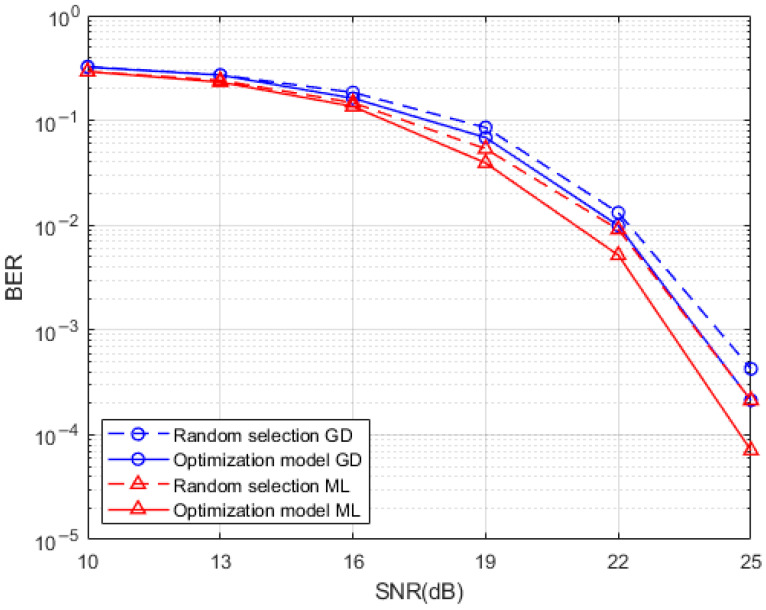
Hamming distance optimized index mapping and random selection mapping performance comparison.

A comparison of the BER performance of the system produced by the Hamming distance optimized index mapping model with the random selection mapping model for a sub-grid group size of 4 with the number of activated sub-grids of 2 and for a sub-grid group size of 8 with the number of activated sub-grids of 3 is presented in Figure 7 and Figure 8, respectively. It can be seen from the simulations that the use of the Hamming distance optimization model for the redundancy in the indexed combinatorial sequences brings a degree of optimization to the BER performance of the system. This optimization effect is more obvious at high SNR, which is reflected by both the ML detection algorithm and the GD detection algorithm at the system receiver. The BER performance improvement of the Hamming distance optimization model is more pronounced at the receiver side when the SNR is higher than 15dB while using the ML detection algorithm and the GD detection algorithm. This is due to the fact that the signal is more heavily disturbed by noise at low SNR which interferes with the determination of the location of the active sub-grid during demodulation. By comparing the performance of two different detection algorithms when the index combination mapping model is optimized by the Hamming distance, the effect of the GD detection algorithm in the optimization of the index combination mapping model under the condition of high SNR is almost the same as that of the ML detection algorithm without the index mapping model. This means that a relatively good BER performance can be achieved at the same time when a relatively low complexity detection algorithm is used, and the system BER performance can be further improved when a relatively high complexity ML detection algorithm is adopted.

## 5. Conclusions

This paper proposes an OTFS-IM underwater acoustic communication system based on Hamming distance optimization, which introduces index modulation into the DD domain. The performance of the OTFS-IM underwater acoustic communication system is further improved by the proposed Hamming distance optimization model to eliminate the redundancy present in the indexed combinatorial sequence. The proposed underwater acoustic communication system and Hamming distance optimized index mapping model were verified by varying the size of the sub-grid groups and the number of activated sub-grids in them and by using different detection methods at the receiver side. The results show that the proposed Hamming distance optimization model optimizes the BER performance of the OTFS-IM underwater acoustic communication system and further enhances the robustness of the OTFS-IM underwater acoustic communication system. Combined with the fact that the OTFS-IM underwater acoustic communication system has the ability to control DD domain sub-grid selection activation more flexibly than conventional OTFS systems, this feature enables OTFS-IM systems to achieve a better compromise between transmission reliability and SE in time-varying channels. Therefore, it can provide a more flexible and reliable solution for underwater acoustic communication systems.

## Figures and Tables

**Table 1:** Index mapping table for $\left(n,k\right)=\left(4,2\right)$.

Index Bit	Active Sub-Grid	Sub-Grid Group
$\left[\begin{array}{cc} 0 & 0 \end{array}\right]$	$\left[\begin{array}{cc} 1 & 2 \end{array}\right]$	${\left[\begin{array}{cccc} s_{\beta ,1} & s_{\beta ,2} & 0 & 0 \end{array}\right]}^{T}$
$\left[\begin{array}{cc} 0 & 1 \end{array}\right]$	$\left[\begin{array}{cc} 1 & 3 \end{array}\right]$	${\left[\begin{array}{cccc} s_{\beta ,1} & 0 & s_{\beta ,2} & 0 \end{array}\right]}^{T}$
$\left[\begin{array}{cc} 1 & 0 \end{array}\right]$	$\left[\begin{array}{cc} 2 & 4 \end{array}\right]$	${\left[\begin{array}{cccc} 0 & s_{\beta ,1} & 0 & s_{\beta ,2} \end{array}\right]}^{T}$
$\left[\begin{array}{cc} 1 & 1 \end{array}\right]$	$\left[\begin{array}{cc} 3 & 4 \end{array}\right]$	${\left[\begin{array}{cccc} 0 & 0 & s_{\beta ,1} & s_{\beta ,2} \end{array}\right]}^{T}$

**Table 2:** Index mapping table for Hamming distance optimization.

Index Bit	Active Sub-Grid	Sub-Grid Group
$\left[\begin{array}{cc} 0 & 0 \end{array}\right]$	$\left[\begin{array}{cc} 1 & 2 \end{array}\right]$	${\left[\begin{array}{cccc} s_{\beta ,1} & s_{\beta ,2} & 0 & 0 \end{array}\right]}^{T}$
$\left[\begin{array}{cc} 0 & 1 \end{array}\right]$	$\left[\begin{array}{cc} 1 & 3 \end{array}\right]$	${\left[\begin{array}{cccc} s_{\beta ,1} & 0 & s_{\beta ,2} & 0 \end{array}\right]}^{T}$
$\left[\begin{array}{cc} 1 & 0 \end{array}\right]$	$\left[\begin{array}{cc} 2 & 4 \end{array}\right]$	${\left[\begin{array}{cccc} 0 & s_{\beta ,1} & 0 & s_{\beta ,2} \end{array}\right]}^{T}$
$\left[\begin{array}{cc} 1 & 1 \end{array}\right]$	$\left[\begin{array}{cc} 3 & 4 \end{array}\right]$	${\left[\begin{array}{cccc} 0 & 0 & s_{\beta ,1} & s_{\beta ,2} \end{array}\right]}^{T}$
redundancy	$\left[\begin{array}{cc} 2 & 3 \end{array}\right]$	${\left[\begin{array}{cccc} 0 & s_{\beta ,1} & s_{\beta ,2} & 0 \end{array}\right]}^{T}$
redundancy	$\left[\begin{array}{cc} 1 & 4 \end{array}\right]$	${\left[\begin{array}{cccc} s_{\beta ,1} & 0 & 0 & s_{\beta ,2} \end{array}\right]}^{T}$

**Table 3:** Time-varying underwater acoustic channel parameters.

Parameters	Value
Surface height (m)	100
Transmitter height (m)	20
Receiver height (m)	50
Channel distance (m)	1000
Spread factor (kHz)	1.7
Doppler factor (v/c)	10^−4^

## Data Availability

The data presented in this paper are available after contacting the corresponding author.
